# Integrated Food Chain Surveillance System for *Salmonella* spp. in Mexico^1^

**DOI:** 10.3201/eid1403.071057

**Published:** 2008-03

**Authors:** Mussaret B. Zaidi, Juan Jose Calva, Maria Teresa Estrada-Garcia, Veronica Leon, Gabriela Vazquez, Gloria Figueroa, Estela Lopez, Jesus Contreras, Jason Abbott, Shaohua Zhao, Patrick McDermott, Linda Tollefson

**Affiliations:** * Hospital General O’Horan, Mérida, Mexico; † Instituto Nacional de Ciencias Medicas y Nutricion, Salvador Zubiran, Mexico City, Mexico; ‡ Centro de Investigacion y Estudios Avanzados del Instituto Politécnico Nacional, Mexico City, Mexico; § Secretaría de Salud del Estado de Michoacan, Morelia, Mexico; ¶ Laboratorio Estatal de Salud Pública y Hospital Central, Servicios de Salud del Estado de San Luis Potosí, San Luis Potosi, Mexico; # Hospital Infantil del Estado de Sonora, Hermosillo, Mexico; ** Food and Drug Administration, Laurel, Maryland, USA; †† Food and Drug Administration, Rockville, Maryland, USA

**Keywords:** Salmonella, surveillance, food chain, antimicrobial resistance, PFGE, Mexico, research

## Abstract

Few developing countries have foodborne pathogen surveillance systems, and none of these integrates data from humans, food, and animals. We describe the implementation of a 4-state, integrated food chain surveillance system (IFCS) for *Salmonella* spp. in Mexico. Significant findings were 1) high rates of meat contamination (21.3%–36.4%), 2) high rates of ceftriaxone-resistant *S*. Typhimurium in chicken, ill humans, and swine (77.3%, 66.3%, and 40.4% of *S*. Typhimurium T isolates, respectively), and 3) the emergence of ciprofloxacin resistance in *S*. Heidelberg (10.4%) and *S*. Typhimurium (1.7%) from swine. A strong association between *Salmonella* spp. contamination in beef and asymptomatic *Salmonella* spp. infection was only observed in the state with the lowest poverty level (Pearson r = 0.91, p<0.001). Pulsed-field gel electrophoresis analysis of 311 *S*. Typhimurium isolates showed 14 clusters with 102 human, retail meat, and food-animal isolates with indistinguishable patterns. An IFCS is technically and economically feasible in developing countries and can effectively identify major public health priorities.

## Abstract 2

This system effectively identified major public health priorities.

Diarrheal diseases are leading causes of childhood illness and death in developing countries (*1*). Many of these infections are acquired through contaminated food and water, but the fraction attributable to each food category is unknown. In the face of worldwide increase in antimicrobial-resistant foodborne pathogens (FBP) and the growing globalization of travel and food trade, the World Health Organization has recommended the establishment of national networks that conduct surveillance along the entire food chain (*2*). Industrialized nations have implemented FBP surveillance systems that include a combination of passive, active, and outbreak data sources (*3*,*4*). In certain countries, outbreak data constitute a considerable proportion of human surveillance (*4*).

In developing countries, information on food-borne disease is scant. Passive surveillance systems are generally inadequate because 1) patients with diarrhea do not seek medical attention; 2) appropriate samples are not sent for culture; or 3) physicians may not report culture-based cases, including deaths, to a public health reference center. Similarly, outbreak information is frequently unsubstantial, either because health authorities lack the capabilities or resources for detection, or presumably, because diarrheal diseases are highly endemic and outbreaks may be less common or obvious than in industrialized countries. Furthermore, comparability between human, food, and animal data is hindered by the lack of standardized laboratory methods and the absence of joint collaboration between the medical, food, and veterinary sectors.

Integrated food chain surveillance systems (IFCSs) are presently established in only a few industrialized countries. Before this study, national data on FBD in Mexico were based on the syndromatic diagnosis of diarrhea. Beginning in 2002, we established an IFCS for *Salmonella* spp., *Campylobacter* spp.*,* and *Escherichia coli* in 4 states from geographically different regions. The system enabled us to identify important human health risks and to establish future research and public health priorities. This article summarizes the data on *Salmonella* spp. obtained from March 2002 through August 2005.

## Materials and Methods

### Study Setting and Epidemiologic Design

The food safety authorities in Mexico (Sistema Federal Sanitario, Secretaría de Salud) divide the country’s 32 states into 5 regions. The 4 states included in this network belong to and are representative of each of the 4 largest regions as follows: Sonora (Region I, Northwest), San Luis Potosi (Region II, Gulf-Central), Michoacan (Region III, Central-Pacific), and Yucatan (Region V, Southeast). In all states, food-animal production is a major economic activity, and most of the circulating retail meat is locally produced. Active surveillance was initiated in 2002 with samples from ill and asymptomatic persons and retail pork, chicken, and beef. Collection of food-animal intestines from chicken, swine, and cattle at slaughterhouses was initiated in mid-2003. The number of samples from food animals and retail meat was designed to reflect regional consumption of each meat product and was limited to domestic production only. In each state, 1 city was sampled per month. Cities with large populations were sampled on repeated occasions; however, different retail outlets and kindergartens were selected for each sampling session. The sampling scheme was designed to follow the food chain in a temporal fashion. Food-animal intestines were collected at each municipal slaughterhouse on day 1, followed by raw retail meat on days 2–4, and fecal samples from asymptomatic kindergarten children on days 7–14. To make the samples in each city as representative as possible, meat was purchased from at least 3 different retail outlets, and only those kindergartens with >40 children were selected for the study. Samples from ill children (which included those with severe and moderate diarrhea as well as those with systemic infections) were collected at the major state government hospitals through active surveillance.

Network activities were incorporated into existing programs at each state health department to increase the likelihood of long-term sustainability of the IFCS. Department inspectors conducted the slaughterhouse surveillance, and diarrheal diseases were monitored at the oral rehydration units and pediatric emergency services that participated in national programs for acute enteric diseases.

### Study Definitions and Ethical Considerations

Case definitions for diarrhea and asymptomatic children have been described (*5*). Socioeconomic indicators previously used to measure poverty (*6*), such as literacy, household sanitary infrastructure, and income, were obtained from recent national population surveys (*7*,*8*). The internal review boards and ethics committees at all participating institutions approved the protocol, and written informed consent was collected from all participants or their guardians to obtain fecal samples and use the clinical and microbiologic information for scientific studies.

### Microbiologic Methods

Participating laboratories used the same standardized methods for isolating and identifying *Salmonella* spp. from human and nonhuman specimens (*9*). Biochemically confirmed isolates were serotyped according to the Kaufmann-White scheme (*10*). All isolates were tested for susceptibility to ampicillin, chloramphenicol, ceftriaxone, ciprofloxacin, gentamicin, kanamycin, nalidixic acid, streptomycin, sulfisoxazole, trimethoprim-sulfamethoxazole, and tetracycline by disk diffusion according to standard guidelines (*11*). External quality control was performed twice a year by the coordinating center in Yucatan. MICs for ceftriaxone, ciprofloxacin, and nalidixic acid were determined by agar dilution (*12*). For purposes of this study, the terms “resistant” or “resistance” refer to strains with zone diameters below or MICs above the susceptible breakpoint. For ciprofloxacin, resistance was defined as an MIC >2 μg/mL. Because isolates with an MIC to ciprofloxacin of 0.12–1.0 μg/mL have been associated with therapeutic failure (*13*), they were included in the analysis and are referred to in this article as “decreased susceptibility.” Isolates that were resistant to ceftriaxone (MIC >16) were further tested for susceptibility to piperacillin, ticarcillin, aztreonam, cefoxitin, ceftazidime, cefotaxime, ceftiofur, cefepime, and imipenem, and also screened for extended spectrum β-lactamases by disk diffusion using ceftazidime and cefotaxime with and without clavulanic acid (*11*). Data analysis was performed with Whonet software version 5.3 (www.who.int/drugresistance/whonetsoftware/en).

### Pulsed-field Gel Electrophoresis

Because of its capacity for virulence and multidrug resistance, *S.* Typhimurium isolates were selected for pulsed-field gel electrophoresis (PFGE) analysis to determine genetic relatedness among human, retail meat, and food-animal isolates from all 4 states. PFGE was performed according to the standard protocol developed by the Centers for Disease Control and Prevention (*14*), which used digestion by *XbaI*. Results were analyzed by using the BioNumerics Software version 3.0 (Applied-Maths, Kortrijk, Belgium), and banding patterns were compared by using Dice coefficients with a 1.5% band position tolerance. A cluster was defined as a group of >2 strains that shared a unique PFGE restriction pattern.

### Statistical Methods

The χ^2^ test was used for comparison of proportions, with an α value of 0.05. Association between type of infection (diarrhea-associated vs. asymptomatic) and the isolation of an extended-spectrum cephalosporin (ESC)–resistant *S*. Typhimurium were analyzed in a 2 × 2 table, and the odds ratio (OR) and its 95% confidence interval (CI) were calculated. Associations between the median rates of *Salmonella* spp. in retail chicken, pork, and beef and the median percentage of asymptomatic *Salmonella* spp. infection in children for each city were calculated with Pearson’s correlation coefficient (r) using SPSS software version 10.0 (SPSS Inc., Chicago, IL, USA).

## Results

### Epidemiologic Surveillance

**Figure 1:**
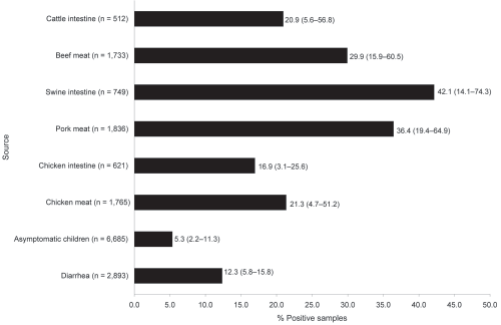
Percentage of human, retail meat, and food-animal samples positive for *Salmonella* spp. detected by an integrated food chain system in Mexico, 2002–2005. Numbers to the right of bars indicate average values, and numbers in parentheses indicate the frequency of positive samples in the states with the lowest and highest prevalence, respectively. The number of specimens examined from each source (n) is shown next to each source heading.

Figure 1 shows the number of specimens sampled from each source and the percentage that were positive for *Salmonella* spp. The prevalence of *Salmonella* spp. was highest in swine intestines and pork meat (42.1% and 36.4%, respectively), followed by cattle intestines and beef (20.9% and 29.9%, respectively), and chicken intestines and chicken meat (16.9% and 21.3%, respectively). *Salmonella* was isolated from 12.3% of hospital-samples from children with cases of diarrhea and 5.3% of asymptomatic kindergarten children.

Socioeconomic indicators and the rates of meat contamination and human *Salmonella* infections for each state are compared in Table 1. Socioeconomic levels for each state were graded on the basis of the 4 indicators shown in the table; Yucatan was classified as the state with the lowest level, followed by San Luis Potosi, Michoacan, and Sonora (highest level). One hundred forty-one sampling sessions were conducted in 64 cities. Samples from all 3 retail meats and kindergarten children were available for 61 cities in the 4 states for correlation analysis. In 11 cities in the state of Sonora, a strong direct correlation was found between each city’s prevalence of beef contamination and its prevalence of asymptomatic *Salmonella* spp. infection (r = 0.91, p<0.001). A moderate correlation was found between pork meat and asymptomatic infection (r = 0.62, p = 0.04). No association was observed for retail meat contamination and human asymptomatic infection in the cities from the other 3 states.

**Table 1:** Comparison of socioeconomic indicators and prevalence of retail meat contamination and human *Salmonella* infection, by state, Mexico, 2002–2005

Indicator	Yucatan, %	Sonora, %	San Luis Potosi, %	Michoacan, %
Population >15 y illiterate or with incomplete primary education	40.1	24.0	37.1	44.0
Households with no toilet or latrine	24.6	7.0	14.0	15.4
Households with no sewage system	40.8	20.2	37	24.4
Working population earning <$4 US/d	23.4	6.7	16.4	13.0
Average prevalence of *Salmonella* in retail meat	59.1	14.2	29.7	16.0
Average prevalence of *Salmonella* in diarrheal episodes	15.8	12.6	10.8	5.8
Average prevalence of *Salmonella* in asymptomatic children	11.3	4.4	2.2	1.9

*S*. Typhimurium and *S*. Enteritidis were the top 2 serovars isolated from ill humans (22.2% and 14.5%, respectively). Among food animals, swine were the most important reservoir of *S*. Typhimurium (10.2% of *Salmonella* spp. isolates), and chickens were the main reservoir of *S*. Enteritidis (11.9% of *Salmonella* spp. isolates) (Table 2). Both humans and animals harbored a considerable diversity of serovars, ranging from 47 to 56 serovars among the different sources. A total of 392 isolates were collected from clinically ill humans. Of these, 26 were isolated from patients with bacteremia and meningitis; 20 (77%) of these isolates were *S*. Typhimurium (8), *S*. Enteritidis (6), and *S.* Typhi (6).

**Table 2:** *Salmonella* serovars in ill humans and their relative frequency in asymptomatic children and food animals, Mexico, 2002–2005

Serovar	% for each serovar relative to the total no. of *Salmonella* serovars				
	Ill humans* (n = 392)	Asymptomatic children (n = 373)	Chicken† (n = 546)	Swine† (n = 1237)	Cattle† (n = 767)
Typhimurium	22.2	6.7	4.6	10.2	6.8
Enteritidis	14.5	3.2	11.9	0.1	0.1
Agona	6.6	8.3	9.5	9.3	7.3
Muenchen	5.1	3.5	0.6	1.9	2.5
Oranienburg	4.1	4.3	0.9	0.3	0.3
Anatum	3.8	8.0	4.8	13.0	17.7
Newport	3.8	5.4	0.0	0.7	2.6
Meleagridis	3.1	6.4	5.3	11.6	13.0
Other	36.8	54.2	62.4	52.9	49.7

*Ill humans include 366 isolates from enteric infections and 26 isolates from invasive infections. †Data for food animal intestine and the corresponding retail meat have been combined because serovars were very similar.

### Antimicrobial Drug Resistance

The percentages of isolates from human and food-animal sources that were resistant to antimicrobial agents are given in Table 3. Antimicrobial drug resistance was highest in ill humans and swine. Resistance to nalidixic acid was highest in *S.* Albany (57%, 65/115) and *S*. Enteritidis (53%, 36/68) from chicken and in *S*. Typhimurium (74%, 133/180) and *S*. Anatum (31%, 92/294) from swine and cattle. Resistance to ciprofloxacin emerged in 2003 in *S*. Heidelberg (10.4%, 5/48) and *S*. Typhimurium (1.7%, 2/127) from swine. Decreased susceptibility to ciprofloxacin was detected in 16.4% of all *Salmonella* spp. isolates (545/3,315) and was most commonly found in chickens (24.9% of all isolates from source), followed by swine (18% of all isolates from source) and ill humans (17.3% of all isolates from source). ESC resistance was first detected in serovar Typhimurium in 2002. From 2002 to 2005, ESC resistance increased from 1.6% to 4.9% and was detected in 6 other serovars. Isolates resistant to ceftriaxone were also resistant to piperacillin, ticarcillin, cefoxitin, ceftazidime, cefotaxime, ceftiofur, and aztreonam and did not show increased susceptibility in the presence of clavulanic acid, which suggests the presence of an AmpC-like β-lactamase. ESC resistance was highest in *S*. Typhimurium (42% of all ST isolates, 132/314), followed by *S.* Bredeney (7.1%, 1/14), *S*. Newport (6.3%, 4/64), *S*. Reading (2.4%, 2/85), *S*. Uganda (2.4%, 1/42), *S*. Kentucky (2.2%, 1/46), and *S*. Anatum (0.5%, 2/365).

**Table 3:** Antimicrobial drug resistance in *Salmonella* isolates from humans, retail meat, and food animals in Mexico, 2002–2005*

Source	% Resistant†										
	AMP	CHL	CIP	CRO	GEN	KAN	NAL	STR	SU	SXT	TET
Ill humans (n = 392)	25.5	23.4	0.0	14.5	11.7	11.2	24.6	61.1	49.7	24.3	41.2
Asymptomatic children (n = 373)	7.8	8.4	0.0	1.4	2.2	0.5	8.6	45.9	35.4	8.0	26.1
Chicken‡ (n = 546)	7.7	7.4	0.0	3.6	2.6	1.8	30.6	58.9	38.9	11.5	36.8
Swine‡ (n = 1,237)	18.3	22.9	0.6	4.2	8.4	9.0	26.0	73.1	62.1	24.2	55.3
Cattle‡ (n = 767)	11.9	14.1	0.0	1.2	6.6	7.2	20.8	71.6	53.1	19.2	48.8

*AMP, ampicillin; CHL, chloramphenicol; CIP, ciprofloxacin; CRO, ceftriaxone; GEN, gentamicin; KAN, kanamycin; NAL, nalidixic acid; STR, streptomycin; SU, sulfisoxazole; SXT, trimethoprim-sulfamethoxazole; TET, tetracycline. †Includes resistant and intermediate. ‡Includes isolates from food-animal intestines and the corresponding retail meat.

*S*. Typhimurium showed particularly high antimicrobial-drug resistance rates in both humans and food animals (Table 4). High resistance rates to ESCs were observed in poultry (77.3%), ill humans (66.3%), and swine (40.4%); multidrug resistance to >4 antimicrobial drug classes was found in 86.6% of isolates. ST isolated from an ill patient was 6 times more likely to be ESC-resistant than isolates from asymptomatic children (OR 6.3, 95% CI 2.3–17.6; χ^2^ 14.4, p<0.001).

**Table 4:** Antimicrobial drug resistance in *Salmonella enterica* serovar Typhimurium isolates from humans, retail meat and food animals in Mexico, 2002–2005*

Source	% Resistant†										
	AMP	CHL	CIP	CRO	GEN	KAN	NAL	STR	SU	SXT	TET
Ill humans (n = 87)	79.3	80.5	0.0	66.3	44.8	33.3	55.1	97.3	91.9	71.2	88.5
Asymptomatic children (n = 25)	36.0	36.0	0.0	25.0	4.0	4.0	12.0	73.9	60.0	24.0	44.0
Chicken‡ (n = 22)	100.0	86.4	0.0	77.3	18.2	18.1	27.2	100.0	90.9	54.5	90.9
Swine‡ (n = 127)	61.9	88.9	1.7	40.4	47.6	41.3	72.2	97.2	92.9	63.5	94.4
Cattle‡ (n = 53)	47.2	71.7	0.0	7.5	45.3	35.9	79.2	94.6	90.6	56.6	92.5

*AMP, ampicillin; CHL, chloramphenicol; CIP, ciprofloxacin; CRO, ceftriaxone; GEN, gentamicin; KAN, kanamycin; NAL, nalidixic acid; STR, streptomycin; SU, sulfisoxazole; SXT, trimethoprim-sulfamethoxazole; TET, tetracycline. †Includes resistant and intermediate. ‡Includes isolates from food-animal intestines and the corresponding retail meat.

### PFGE

**Figure 2:**
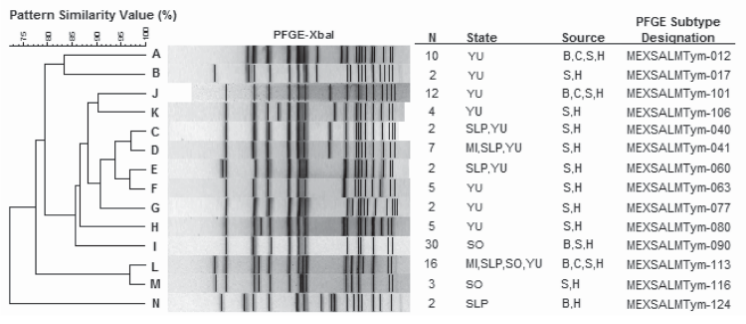
Selected pulsed-field gel electrophoresis (PFGE) clusters that represent 102 strains of *Salmonella* Typhimurium and shared indistinguishable PFGE patterns among humans (H), chicken meat and intestine (C), pork meat and swine intestine (P), and beef meat and cattle intestine (B). Several clusters (C,D, E, and L) were present in more than one state. MI, Michoacan; SLP, San Luis Potosi; SO, Sonora; YU, Yucatan. An expanded version of this figure containing the complete set of PFGE patterns is available from http://www.cdc.gov/EID/content/14/3/429-G2.htm.

The network collected 314 *S*. Typhimurium isolates, of which 311 were available for PFGE (Figure 2). A total of 126 PFGE patterns were identified. Fourteen clusters (boxes A–N), comprising a total of 102 strains (37 human, 37 retail meat, and 28 food-animal isolates), were common to both humans and food animals. Three patterns (012, 101, 113) were common to humans, and all 3 food animals, and 1 pattern (113) was found in all 4 states. For each state, we found clusters of human isolates that were indistinguishable or closely related to those found in retail meat, food animals, or both.

## Discussion

Our experience shows that the establishment of an IFCS is technically and economically feasible in a developing country such as Mexico. The system effectively identified the serovars that caused the greatest effects of disease as well as major animal reservoirs of these serovars in a setting where *Salmonella* spp. infections are highly endemic and passive surveillance is inadequate. The epidemiologic design also corrected constraints present in other monitoring systems, such as the temporal and spatial dissociation of human, food, and animal isolates, as well as the lack of uniform laboratory methods.

Furthermore, the system yielded epidemiologically meaningful data that provided evidence of the magnitude of *Salmonella* spp. as a health risk. Our average prevalence of *Salmonella* spp. contamination in retail meats (21.3%–36.4%) and the high frequency of human *Salmonella* spp. infection, in conjunction with PFGE clusters of geographically and temporally related human and food-animal isolates, led us to conclude that food animals are a major source of salmonellosis in Mexico. A proportion of these clusters may constitute undetected outbreaks; however, this can only be determined once reliable baseline data have been obtained.

Higher rates of meat contamination were observed in those states with higher poverty levels. This finding can likely be explained by the multiple inadequacies in the sanitary infrastructure that lead to increased contamination and dissemination of FBP throughout the environment, in particular, along the food chain. Interestingly, in Sonora, the state with the lowest poverty level that borders the United States, cities with high rates of beef contamination (and to a lesser degree, pork contamination) also had high rates of asymptomatic carriage of *Salmonella* spp. among children. Sonora is one of the major beef- and pork-producing states in the country, and these retail meats are consumed more frequently than chicken by the local population, which might explain the absence of an association with the latter. Conversely, those cities with low rates of meat contamination had lower rates of asymptomatic carriage. Such associations, however, were not found in the other 3 states with higher poverty levels. The conditions in Sonora may more closely resemble the transmission of *Salmonella* spp. infections in industrialized countries, where most infections are acquired through the food chain (*15*), whereas in the other states *Salmonella* spp. infections are probably acquired by other modes of transmission aside from contaminated food, such as from person to person or by contact with animal feces. In settings with greater fecal-oral transmission, asymptomatic infections would not directly reflect contamination rates in the retail meat. Our findings suggest that cattle, swine, and chicken are important, but not unique, reservoirs of *Salmonella* spp. in Mexico. Other animals not included in this study may contribute to an important proportion of these infections, and therefore, other food categories should be included in the surveillance system in the future.

The high rates of ESC resistance and the emerging resistance to fluoroquinolones are other important public health risks detected by the system. Our increasing resistance rates are consistent with a worldwide upsurge of multidrug-resistant nontyphoidal *Salmonella* spp. (*16*,*17*). Multidrug resistance in our *S*. Typhimurium isolates is the most pressing concern. *bla*_CMY-2_ *S.* Typhimurium is now a major public health problem in Yucatan, where its prevalence increased from 0% in 2000 and 2001 to 75% in 2004 and 2005 and where it has caused fatal infections in young infants (*18*). The higher rate of ESC resistance in ill children with *S*. Typhimurium infection than in asymptomatic children has 2 possible explanations. The first is that these children received antimicrobial drugs shortly before their diarrheal episode, a known risk factor for acquiring multidrug-resistant *Salmonella* spp. infection (*19*,*20*). A second possibility propounded by other investigators is that antimicrobial drug–resistant *Salmonella* spp. is associated with increased virulence (*21*,*22*). We believe that the higher prevalence of ESC resistance in our ill children is of substantial public health importance and that the mechanisms leading to this increased resistance should be further investigated.

Because the network is in its early stages, it does not yet have the capabilities of a mature surveillance system. At present, it is not designed to measure the human health impact of contaminated food consumption or to perform cost-benefit analyses of prevented deaths or reduced days of hospitalization. Furthermore, it does not have a high sensitivity for outbreak detection. All of these capabilities need to be developed in the future. The main objective of the initial phase of the IFCS (2002–2005) was to collect baseline data with good internal validity. Enormous effort was devoted to training staff and achieving good laboratory quality control. During subsequent phases, priority should be given to measuring illness, proportion of deaths, and the economic impact of salmonellosis. This goal could be achieved by increasing the number of sentinel hospitals per state and organizing the systematic collection of clinical and cost-related data. Furthermore, future surveillance should include animal farms and data on local food consumption and antimicrobial usage. Lastly, due to its nature as an early stage study, the system yielded predominantly descriptive information. These data have served to generate several hypotheses that can be tested in future research.

The network described in this study included 4 representative states and was originally established in collaboration with state health authorities. It is currently being transferred to the federal food safety authorities. Based on this study, we believe that a sustainable IFCS could include a 5-state network (one from each representative region). Operating costs (laboratory supplies and staff salaries) for this system, including PFGE analysis for the top 2 serotypes found in ill children, would run US $500,000 per year, which is equivalent to ≈2% of the Federal Sanitary System’s yearly budget (*23*).

In conclusion, this project can serve as a model for developing countries to establish an IFCS. We believe that, in the initial stages, efforts should focus on training of staff; laboratory quality control; and achieving good integration of human, food, and animal data. Once this stage has been consolidated and good baseline data have been obtained, the system can become increasingly complex according to needs and available resources. In a period of 3 years and with a modest financial investment, our system was able to identify several important emerging public health threats that should be the focus of future evidence-based interventions. The reduction of *Salmonella* spp. contamination throughout the food chain, as well as the prevention and control of ESC-resistant and fluoroquinolone-resistant *Salmonella* spp. in food animals and retail meats, should receive major priority. The striking differences between the various states can be used by policy makers to design interventions and allocate resources by region to reduce the effects of FBD in those areas with the greatest risk. Finally, control measures instituted throughout the food chain should be linked to other national programs that include the use of oral rehydration therapy, the promotion of breast feeding, and improved nutrition as part of a joint effort to reduce illness and death from diarrheal disease (*24*,*25*).
